# A Novel Therapeutic Approach in the Treatment of Pulmonary Arterial Hypertension: *Allium ursinum* Liophylisate Alleviates Symptoms Comparably to Sildenafil

**DOI:** 10.3390/ijms18071436

**Published:** 2017-07-04

**Authors:** Mariann Bombicz, Daniel Priksz, Balazs Varga, Andrea Kurucz, Attila Kertész, Akos Takacs, Aniko Posa, Rita Kiss, Zoltan Szilvassy, Bela Juhasz

**Affiliations:** 1 Department of Pharmacology and Pharmacotherapy, Faculty of Medicine, University of Debrecen, H-4032 Debrecen, Hungary; bombicz.mariann@pharm.unideb.hu (M.B.); priksz.daniel@pharm.unideb.hu (D.P.); varga.balazs@pharm.unideb.hu (B.V.); kurucz.andrea@pharm.unideb.hu (A.K.); akos.takacs6@gmail.com (A.T.); kissrita74@gmail.com (R.K.); szilvassy.zoltan@med.unideb.hu (Z.S.); 2 Department of Cardiology, Faculty of Medicine, University of Debrecen, H-4032 Debrecen, Hungary; dr.kertesz.attila@gmail.com; 3 Department of Physiology, Anatomy and Neuroscience, Faculty of Science and Informatics, University of Szeged, Kozep Fasor 52, H-6726 Szeged, Hungary; paniko@bio.u-szeged.hu

**Keywords:** *Allium ursinum*, pulmonary arterial hypertension, phosphodiesterase, sildenafil, monocrotaline

## Abstract

Right-sided heart failure—often caused by elevated pulmonary arterial pressure—is a chronic and progressive condition with particularly high mortality rates. Recent studies and our current findings suggest that components of Wild garlic (*Allium ursinum*, AU) may play a role in reducing blood pressure, inhibiting angiotensin-converting enzyme (ACE), as well as improving right ventricle function in rabbit models with heart failure. We hypothesize that AU may mitigate cardiovascular damage caused by pulmonary arterial hypertension (PAH) and has value in the supplementary treatment of the complications of the disease. In this present investigation, PAH was induced by a single dose of monocrotaline (MCT) injection in Sprague-Dawley rats, and animals were divided into 4 treatment groups as follows: I. healthy control animals (Control group); II. pulmonary hypertensive rats (PAH group); III. pulmonary hypertensive rats + daily sildenafil treatment (Sildenafil group); and IV. pulmonary hypertensive rats + Wild garlic liophylisate-enriched chow (WGLL group), for 8 weeks. Echocardiographic measurements were obtained on the 0 and 8 weeks with fundamental and Doppler imaging. Isolated working heart method was used to determinate cardiac functions ex vivo after thoracotomy on the 8th week. Histological analyses were carried out on excised lung samples, and Western blot technique was used to determine Phosphodiesterase type 5 enzyme (PDE5) expression in both myocardial and pulmonary tissues. Our data demonstrate that right ventricle function measured by echocardiography was deteriorated in PAH animals compared to controls, which was counteracted by AU treatment. Isolated working heart measurements showed elevated aortic flow in WGLL group compared to PAH animals. Histological analysis revealed dramatic increase in medial wall thickness of pulmonary arteries harvested from PAH animals, but arteries of animals in sildenafil- and WGLL-treated groups showed physiological status. Our results suggest that bioactive compounds in *Allium ursinum* could have beneficial effects in pulmonary hypertension.

## 1. Introduction

Right-sided heart failure—often caused by pulmonary hypertension resulting from elevated arterial pressure—is a chronic and progressive condition with particularly high mortality rates. Pulmonary arterial hypertension (PAH) is a vascular dysfunction characterized by abnormalities of endothelial and smooth muscle cells of pulmonary vessels. As a result of the processes, vascular resistance increases, which quickly starts depleting compensatory mechanisms in the right ventricle. This maladaptation eventually leads to irreversible damage of the cardiovascular system and premature death of patients suffering from the disease. PAH itself essentially represents a vasculopathy, and a chronic, progressive disease entity. This is reflected in the current etiology and pathophysiology-based clinical classification by the World Health Organization (WHO) of the types of pulmonary hypertension (PH), where PAH represents one individual group [1].

The condition is predominantly characterized by progressively-increasing vascular resistance (PVR) of pulmonary arteries. Since the right ventricle is highly sensitive to changes in afterload, adaptive mechanisms to increased pressure—such as Frank-Starling mechanism and ventricular muscle hypertrophy—appear in early stages of the disease [2]. Increased tension in chordae tendinae and dilatation of the annulus leads to tricuspid regurgitation, resulting in dilation of the right atrium, right ventricular volume overload and decreased cardiac output [3]. Ischemic lesions also appear due to worsening hypethrophy. The interventricular septum becomes thinner and is even pressed into the left side of the heart, thereby reducing the volume of the left ventricle during diastole. The processes necessarily result in decreased cardiac output caused by abnormal reduction of the left ventricular preload. This vicious circle ends in systemic hypoxia, severe heart failure and premature death [4].

The first line of underlying pathological processes in PAH is vasoconstriction of pulmonary arteries caused by an imbalance of vasoconstrictor and vasodilator mediators. Multiple dysregulated signalling pathways have been identified in PAH patients, and a significant portion of disease-specific therapeutic agents have an influence on the course of the disease through these routes, namely the nitric oxide (NO) pathway (I), the prostacyclin (II) and endothelin-1 pathways (III) [5,6,7,8].

Evidences include decreased NO synthase expression and abnormally high arginase enzyme levels in lungs of PAH patients, as well as overexpression of phosphodiesterase type 5 enzyme (PDE5), therefore reduced cyclic guanosine monophosphate (cGMP) levels [9,10]. Further studies have shown decreased levels of prostacyclin (PGI2) and cyclic adenosine monophosphate (cAMP) in the relevant tissues, which is partly explained by the fact that the expression of prostacyclin synthase is abnormally low in the lungs of these patients [11,12].

Endothelin-1, acting on ETA and ETB receptors, is a vasoactive mediator with concominant proliferative effects on smooth muscle cells in the vessel wall. Under physiological conditions, when endothelium is intact, activation of ETB receptors increases the production of NO and PGI2. However, in PAH patients, the ETA receptor pathway overwhelms these effects, causing vasoconstriction by increasing intracellular calcium levels and activation of protein kinase C enzyme (PKC), as well as inducing proliferation via the MAPK (mitogen-activated protein kinase) pathway in smooth muscle cells of pulmonary vessels. Elevated endothelin-1 levels were identified in affected pulmonary tissues, and the peptide is presented in higher concentrations in PAH patients compared to healthy controls. Moreover, strong correlation has been found in the levels of endothelin-1 and the degree of pulmonary hypertension [13,14].

The first modern therapeutic approach based on the previous facts, was to elevate cGMP levels in the lung, since the required vasodilation caused by NO is mediated by cGMP as a secondary messenger. Since therapeutic admininstration of nitric oxide itself by inhalation is possible but very inconvenient, elevation of cGMP level is more feasible by blocking its metabolization through the inhibition of PDE enzymes. Phosphodiesterase type 5 is the dominant enzyme isoform in the lung that converts cGMP into guanosine monophosphate (GMP), and thus blocking this isoenzyme prevents cGMP breakdown and elevates its intracellular levels [15]. PDE5 has also been found to be overexpressed in pulmonary tissue samples of PAH patients. PDE5 inhibitors (PDE5I) increase the activity of NO-cGMP pathways, and thereby promote vasodilation and even have antiproliferative effects on smooth muscle cells of pulmonary vessels. Sildenafil-citrate, the former blockbuster PDE5I for therapy of erectile dysfunction, was approved by the FDA (U.S. Food and Drug Administration) to treat pulmonary hypertension in the year 2005 [16,17]. Sildenafil has clear benefits in PAH, however, the FDA warns about its long-term use in children because of elevated risk of mortality as revealed in the STARTS-2 trial [18].

Wild garlic (*Allium ursinum* L.), a plant distributed widely in Eurasia and also known as bear’s garlic or buckrams, is a popular dietary component, spice and also an element of traditional medicine. Unfortunately, wild garlic, due to the similarity of the leaves, can easily be mistaken with *Colchicum autumnale*, a toxic, colchicine-containing plant, thus some case-studies report rare, but serious poisonings [19]. The herb has a garlic-like scent due to sulfur-containing compounds and also contains high levels of polyphenol derivatives, mostly flavonoid glycosides [20]. The majority of pharmacological properties of the plant extract or liophylisate are comparable to those of the close relative, cultivated garlic (*Allium sativum*), however, some effects of wild garlic are superior or unique, possibly due to the presence of some specific components, such as phytosterols and a galactolipid-derivative (1,2-di-*O*-α-linolenoyl-3-*O*-β-d-galactopyranosyl-sn-glycerol). This component seems to be specific for *Allium ursinum* and may be absent in other Allium species [21].

While a large amount of data is available on the characteristics of *A. sativum*, possible therapeutic effects of *A. ursinum* are poorly studied, and only a few publications have investigated pharmacological properties of this plant. Antiaggregatory effects on human platelets are well-proven [21,22], as well as significant in vitro inhibition of 5-lipoxygenase (5-LOX) and cyclooxygenase (COX) enzymes [23]. Bioactive components of wild garlic have fairly high antioxidant and free radical scavenger activity, mainly due to superoxide-dismutase, catalase and peroxidase activity of the bulb and leaves [24]. Wild garlic extracts containing ajoene, methyl-ajoene, allicin, diallyl-disulfide have also shown marked inhibition on cholesterol biosynthesis in vitro, equivalently to cultivated garlic [25]. *Allium ursinum* preparations reduce blood pressure and inhibit angiotensin-converting enzyme (ACE) in vivo when tested on spontaneously hypertensive (SHR) rats [26,27]. Capability of ACE inhibition has been further evidenced by other studies, where angiotensin-converting-enzyme inhibitor (ACEI) activity of wild garlic was even found to be superior than such effect of regular garlic [25]. Antihypertensive, cholesterol-lowering, and ACEI effects contribute to cardioprotection, and such property of the plant was investigated in a rat model of ischemia/reperfusion injury. Hearts of rats treated with wild garlic extract showed reduced incidence of ventricular fibrillation and tachycardia with decreased ischemic areas in myocardial tissues [28]. In a rabbit model of hypercholesterolemia-induced heart failure, our research team previously demonstrated that supplementation with Wild garlic improves right ventricle systolic function measured by tricuspid annular plane systolic excursion (TAPSE) [29]. Moreover, studies suggest that bioactive saponines and flavonoids of other Allium species (*A. chinese*, *A. cepa*) have phosphodiesterase enzyme (PDE 5A) inhibitor properties [30,31]. According to the aforementioned findings, we hypothesize that bioactive components of *Allium ursinum* may have benefit in the treatment of pulmonary hypertension, due to possible effects on phosphodiesterase enzyme system in myocardial and pulmonary tissues.

## 2. Results

### 2.1. Mass Spectrometry

The quasi molecular ions cationized by potassium and sodium were observed. It can be assumed that, based on the the mass of [M + K]^+^ or [M + Na]^+^ peaks, the following compounds may be present in the plant, as seen in Table 1: kaempferol-3-*O*-rutinoside (at *m*/*z* 617.4 [M + Na]^+^ and *m*/*z* 633.3 [M + K]^+^); quercitrin (at *m*/*z* 471.2 [M + Na]^+^ and *m*/*z* 487.2 [M + K]^+^); juglanin at *m*/*z* 441.4 [M + Na]^+^ and *m*/*z* 457.3 [M + K]^+^; dracorubin (at *m*/*z* 511.2 [M + Na]^+^ and *m*/*z* 527.2 [M + K]^+^); and blumeatin (at *m*/*z* 325.2 [M + Na]^+^, *m*/*z* 341.2 [M + K]^+^ and *m*/*z* 303.1 [M + H]^+^).

Calculations were made on the basis of molar mass of the K and Na adducts. The exact molar mass of each compound was calculated as follows: measured *m*/*z* minus the molar mass of the adducted ion equals the molar mass of the compound.

### 2.2. Echocardiography

All echocardiographic examinations were completed within a 20-min time interval, and all animals managed to recover from the anesthesia with stable heart rates and respiratory frequencies during the whole procedure. As seen in Table 2, Fractional Shortening (FS) and Ejection Fraction (EF) values did not show any changes among groups. Systolic function estimated by measuring mitral annular plane systolic excursions (MAPSE) showed no changes among treatment groups, a result also observed for Heart rate (HR).

Estimation of right ventricle systolic function was attainable by measuring TAPSE (tricuspid annular plane systolic excursion) values. TAPSE parameters of Control animals remained at normal range (TAPSE_Control_: 2.308 ± 0.074 mm), but significant decreases were found in values of PAH group animals (TAPSE_PAH_: 1.697 ± 0.098 mm) compared to the Controls. Values of WGLL-treated and sildenafil-treated animals were elevated in comparison to PAH group (TAPSE_WGLL_: 2.021 ± 0.071 mm, and TAPSE_Sildenafil_: 2.390 ± 0.069 mm, versus TAPSE_PAH_: 1.697 ± 0.098 mm).

Diastolic function of the left ventricle was estimated using Doppler (PW) techniques, by determining E/A ratios at the mitral valve. E/A ratios were unaffected either by monocrotaline-injection, or by sildenafil- or WGLL-treatment after 8 weeks.

### 2.3. Effects of Monocrotaline (MCT) and Treatments on Isolated Left Ventricular Function

**Figure 1:**
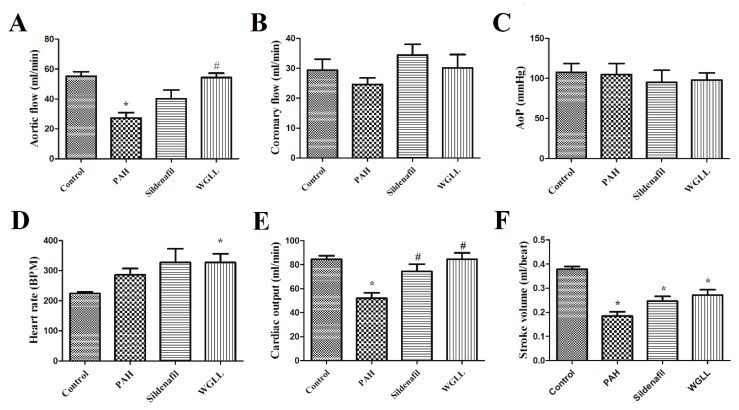
Effects of four treatments on cardiac functions in rats measured ex vivo by isolated working heart method. Treatment groups included Control, PAH (pulmonary arterial hypertension) group, Sildenafil-injected group, and Wild garlic (*Allium ursinum*) liophylisate-enriched chow (WGLL) group. Results are shown as average values for each group of rats ± standard error of the mean (SEM) for aortic flow (AF, mL/min, **A**); coronary flow (CF, mL/min, **B**); aortic pressure (AoP, Hgmm, **C**); heart rate (HR, beat/min, **D**); cardiac output (CO, mL/min, **E**); and stroke volume (SV, mL, **F**). * *p* < 0.05 compared to Control; # *p* < 0.05 compared to PAH. AF, CO and SV values of PAH animals were decreased compared to controls, whereas CO was found to be elevated in sildenafil and WGLL groups compared to PAH group. AF values of WGLL animals were increased in comparison to PAH animals.

Results of LV function obtained by isolated working heart method are shown in Figure 1. Monocrotaline treatment produced reduction in aortic flow (AF) compared to the Control group (AF_PAH_: 27.38 ± 3.447 mL/min vs. AF_Control_: 55.33 ± 2.932 mL/min), and aortic flow of animals after WGLL treatment reached the Control values (AF_WGLL_: 54.36 ± 2.864 mL/min). Coronary flow (CF) and Aortic pressure (AoP) were observed to be unaffected by the treatments (*p* < 0.05). Heart rate of isolated hearts increased in WGLL-treated animals (HR_WGLL_: 327.5 ± 28.22 bpm) in comparison to Control group (HR_Control_: 224.0 ± 5.579 bpm).

### 2.4. Body Mass

We observed no significant differences in body weights among groups, when measured at the endpoint of the study (Control: 485.5 ± 18.40 g; PAH: 502.6 ± 17.79 g; Sildenafil: 443.9 ± 17.10 g; and WGLL: 448.2 ± 10.36 g). A limitation of this study is that the effects of *Allium ursinum* supplementation on food and water consumption were not measured.

### 2.5. Microscopic Morphometry

**Figure 2:**
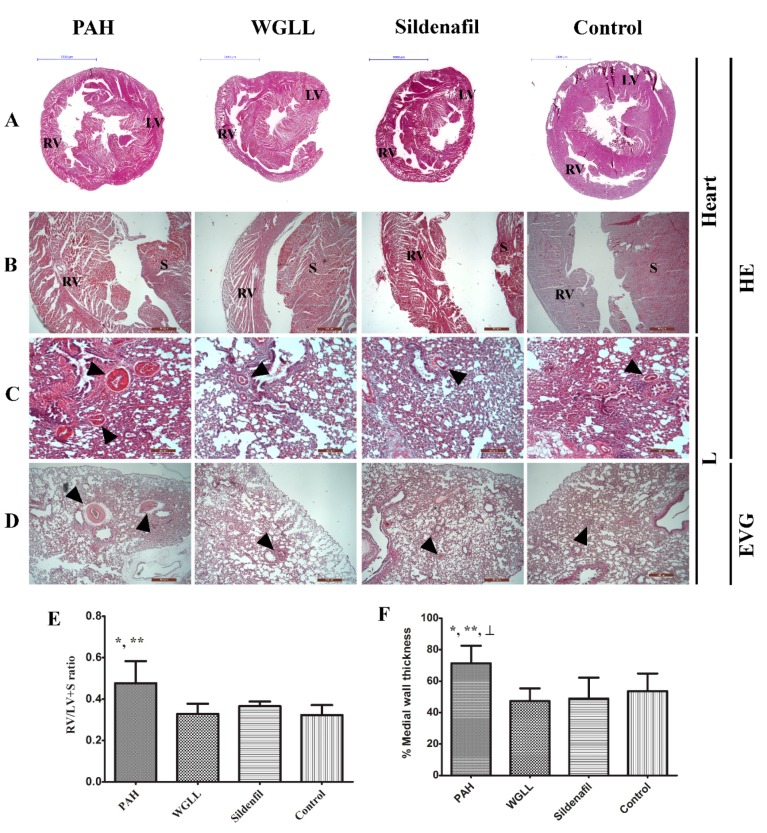
Outcomes of histological analyses of rats receiving four treatments: PAH (pulmonary arterial hypertension), WGLL (Wild garlic (*Allium ursinum*) liophylisate-enriched chow), Sildenafil injections, and Control. Respective figures are: (**A**) Representative images of HE-stained transversal whole heart sections, RV = right ventricle, LV = left ventricle; (**B**) HE staining of the rat hearts from short-axis slice (magnification: 40×); (**C**) Histological examination (HE staining) of lung tissues (magnification: 40×); (**D**) Elastica van Gieson (EVG) staining demonstrates the thickness of pulmonary arterial wall in lung tissues (magnification: 40×); (**E**) Right ventricular hypertrophy (RVH) shown as RV/(LV + S) ratio; and (**F**) Quantitative analysis of peripheral pulmonary arteries demonstrates the medial wall thickness (MWT) in PAH, WGLL, Sildenafil and Control groups. The medial wall thickness was calculated by the equation: % MWT = (M1 + M2)/ED × 100. Dark triangles mark the small pulmonary arteries (**B**–**D**). Lenght of blue scale bars on (**A**): 5000 µm; lenght of red scale bars on (**B**–**D**): 500 µm. Values are expressed as mean ± standard error of mean (SEM; *n* = 15/group). * *p* < 0.05 compared to Control; ** *p* < 0.05 compared to WGLL; ┴ *p* < 0.05 compared to Sildenafil.

Histological examinations of HE-stained heart tissue sections from rats are shown in Figure 2. Right and left ventricle (RV + LV) sections stained with HE showed clear signs of tissue damage in all MCT-treated groups (PAH, WGLL, Sildenafil groups), whereas the Control group is morphologically normal (Figure 2B). Tissue damage in PAH group samples is characterized by contractures of cardiomyocites, hyperbasophilia, hypereosinophilia, wavy arrangement of myofibers, cell edema, lesion and apoptosis of cardiomyocytes. Samples of PAH group show the most dramatic RV hypertrophy in myocardial structure, meanwhile only moderate macroscopic hypertrophy is visible in WGLL-treated RV tissues compared to PAH group. Lung samples of all animals were evaluated by HE- and EVG stains as shown in Figure 2C,D. Right vetricular hypertrophy (RVH) was presented as RV/(LV + S) ratio, as seen in Figure 2E. RV/(LV + S) ratio was increased in PAH group (0.477 ± 0.044) compared to Control (0.323 ± 0.020) and WGLL (0.328 ± 0.020) groups. Medial wall thickness percentage (MWT %) was determined in pulmonary arteries/arterioles (<50 µm diameter) of each group by using the equation: % MWT = (M1 + M2)/ED × 100. Medial wall thickness increased in PAH animals when compared to all other groups (% MWT_PAH_: 71.39 ± 2.628% vs. % MWT_Control_: 53.64 ± 3.240%; % MWT_WGLL_: 47.32 ± 2.084%, and % MWT_Sildenafil_: 48.91 ± 3.444%). We observed no differences in values of WGLL- and Sildenafil-treated animals compared to the Control group (Figure 2F).

### 2.6. Western Blot

**Figure 3:**
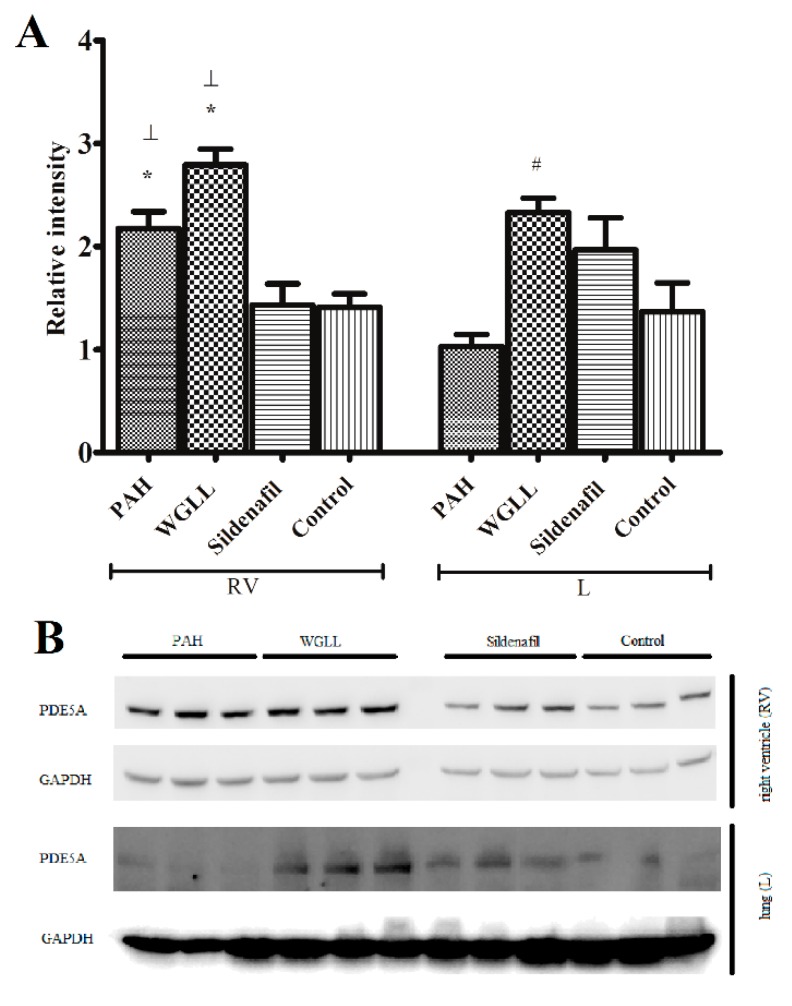
Western blot results from rats receiving four treatments: PAH (pulmonary arterial hypertension), WGLL (Wild garlic (*Allium ursinum*) liophylisate-enriched chow), Sildenafil injection, and Control. (**A**) shows quantified results of Western blot analysis after normalization to Glyceraldehyde 3-phosphate dehydrogenase (GAPDH), and data are presented as mean ± SEM for each treatment group. RV = right ventricle, L = lung (*n* = 9; * *p* < 0.05); and (**B**) shows PDE5A expression in right ventricles (RV) and in lung tissues (L). * *p* < 0.05 compared to Control; # *p* < 0.05 compared to PAH; ┴ *p* < 0.05 compared to Sildenafil.

The levels of PDE5A enzyme expression in right ventricular and lung tis sues are presented in Figure 3A,B. When measured in cardiac tissues, expression of PDE5A in PAH and WGLL groups was elevated compared to levels detected in Sildenafil and Control groups (PAH: 2.174 ± 0.110 and WGLL: 2.690 ± 0.096 vs. Sildenafil: 1.432 ± 0.139 and Control: 1.410 ± 0.088). Additionally, sildenafil treatment showed marked inhibition of PDE5A expression in RV tissue. There were no changes observed in values of WGLL and PAH groups from right vetricle homogenates. In contrast, PDE5 expression levels in MCT-treated lung tissues showed no differences when compared to Control group, however, a significant increase was observed in WGLL in comparison to PAH group animals (Control: 1.367 ± 0.189; WGLL: 2.329 ± 0.094; Sildenafil: 1.968 ± 0.209 vs. PAH: 1.029 ± 0.079; in arbitrary units).

## 3. Discussion

**Figure 4:**
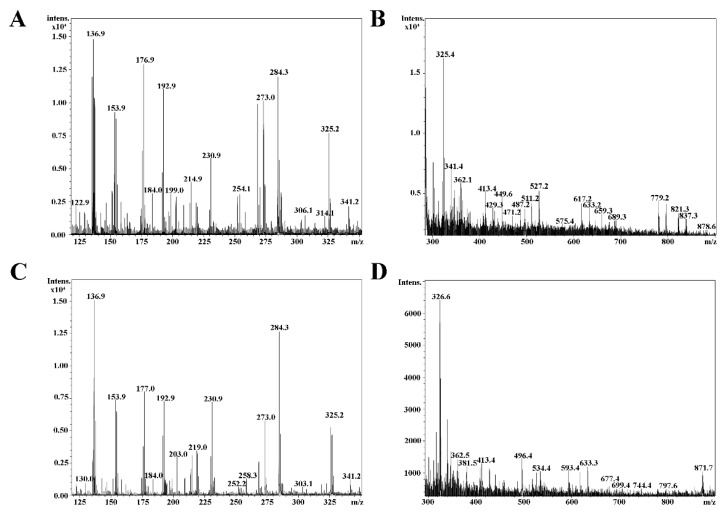
Mass Spectrometry results. MALDI-TOF mass spectra of compounds from *Allium ursinum* leaf liophylisate (WGLL) extracts, using 2,5-Dihydroxybenzoic acid (DHB) matrix. (**A**,**B**) Compounds previously separated by C18 column; (**A**) Compounds with relatively low molecular weight; B: compounds with higher molecular weight; (**C**,**D**) components of WGLL extract, previously separated by silica S100 column; (**C**) Compounds with relatively low molecular weight; (**D**) Compounds with higher molecular weight.

One accomplishment of this present study is characterizing the flavonoid content of *Allium ursinum* leaves, liophylised by the method used for our standard investigations, and comparing it to data available in literature, where chemical constituents were determined from fresh leaves [20]. Our results are well-correlated with previous findings [32], since the dry liophilysate contains several phenolic compounds, i.e., kaempherol derivatives, juglanin, dracorubin, blumetain and quercitrin (as seen in Figure 4 and Table 1); thus we successfully demonstrated that *Allium ursinum* is exceptionally rich in flavonoid compounds. These outcomes have high value, because if a natural product is intended to be used for therapeutic purposes according to evidence-based medicine, investigators need to be aware of its main components and their chemical structure. Furthermore, many pharmacological studies have confirmed the fact that these polyphenols have high potential in prevention and treatment of several disorders [33,34,35].

Echocardiographic data revealed that after 8 weeks of treatment, deterioration was not observed in left ventricular diastolic or systolic functions of pulmonary hypertensive or either WGLL- or sildenafil-treated animals. Tricuspid annular plane systolic excursion (TAPSE) correlates well with right ventricular dysfunction in heart failure patients, and can be easily determined by using M-mode echocardiography. TAPSE parameters were reduced in pulmonary hypertensive rats, but improved after 8 weeks of WGLL treatment. Our findings support the results of other experiments; for instance, Rajkumar et al. showed very similar TAPSE values of MCT-induced PAH rats (around 1.6 mm), as well as healthy animals (above 2 mm) [36]. According to our former and recent findings, *Allium ursinum* liophylisate treatment improves right ventricular function in hypercholesterolemic rabbits [29], as well as in a rat model of MCT-induced pulmonary hypertension. A limitation of this current study is that we failed to consequently visualize pulmonary artery and measure parameters such as pulmonary arterial pressure, acceleration time and pulmonary ejection time by echocardiography, as hallmarks of PAH, formerly described by Thibault et al. [37]. Our disease model was evidenced by other techniques, e.g., measuring medial wall thickness of pulmonary arteries after histological staining.

Isolated working heart is a widespread and well-known method for studying cardiac functions without the innervation of autonomic nervous system [38,39], and our workgroup carried out many experiments based on this method [29,40,41]. Echocardiographic and isolated heart data are often well-correlated in many cardiac parameters, however, discrepancies can also be observed, due to the lack of innervation and vegetative and adaptive reflexes in isolated working heart procedures.

Isolated heart outcomes of the present study (Figure 1) revealed that monocrotaline treatment decreases aortic flow, which characterizes the pump function of the left ventricle. This finding further supports several former outcomes of other authors, where monocrotaline induced PAH [42,43,44], and the consequential right-heart failure eventually leads to left ventricle failure as well [45]. According to our results, sildenafil increased cardiac output when compared to PAH group. Consistent with this, many articles in the scientific literature confirm this finding and verify that sildenafil exerts significant protection in monocrotaline-induced PAH [46,47,48]. Comparably, treatment with WGLL also exerted protection against monocrotaline-induced changes: *Allium ursinum* improved aortic flow and cardiac output compared to the PAH group. Nonetheless, an increase in cardiac output was observed in the WGLL group compared to PAH group; neither heart rate and non stroke volume parameters changed significantly, due to the power of detection. Despite non-significant changes in aortic flow, cardiac output parameters of Sildenafil group compared to PAH followed the same pattern. In a previous work of our team [29], WGLL treatment showed similar effects on isolated heart functional parameters of hypercholesterolemic rabbits after ischemia/reperfusion injury.

Improvements of left or right ventricular function and aortic flow after WGLL treatment have been shown on two animal disease models by our team so far, however, underlying molecular mechanisms, as they seem to be independent from PDE5 system, still require further investigations. Although this study provides useful background information about the effects of WGLL-supplementation on PDE system, measuring endothelial nitric oxide synthase (eNOS) activity or cGMP levels would provide further insights into the potential mechanisms of the protective effect of garlic leaves, which will be addressed in future studies [49].

In this current study, values of aortic pressure did not show any changes among the treatment groups. As this method utilizes an isolated organ, only the most severe left ventricle heart failure could cause aortic pressure changes, i.e., when the myocardium fails to produce the sufficient pressure on the blood in the ventricle to pump it out [50]. In our current study and experimental conditions, maybe due to the relatively short time period, the left ventricle failure was not in this developmental stage, thus we did not observe any changes in this parameter.

The heart rate measurements during isolated heart experiments showed a moderate increase in all of the monocrotaline-treated groups. Such mild increase in heart rate due to monocrotaline treatment has been shown by other authors as well [51].

In our isolated working heart experiments, *Allium ursinum* proved its protecting efficacy again, comparably to sildenafil in a rat model of PAH: both sildenafil and WGLL treatments improved cardiac output values compared to the monocrotaline-alone treated group. Furthermore, in the case of cardiac output, neither of the two differed from values of the healthy control group. Similarly, *Allium ursinum* has been shown to be cardioprotective against other, different types of injuries as well, such as ischemia-reperfusion induced injury [28], and elevated blood pressure [27], thus we consider wild garlic as a possibly valuable supplement in the therapy of various heart diseases.

Although it has been reported frequently that MCT damages pulmonary endothelial cells, the exact toxicological mechanisms by which MCT initiates lung toxicity are still obscure, furthermore, several investigators dispute the relevance of MCT-model in imitating pathobiological processes of the human disease [52]. Despite this fact, MCT-induced pulmonary arterial hypertension is still the most favored model in preclinical research in testing novel therapies; moreover, almost all animal models are imperfect in some way in imitating the patomechanism or manifestation of this disease.

In this work, we demonstrated that MCT-induced pulmonary arterial hypertension caused RV hypertrophy in animal models, a finding published in previous studies as well [53,54]. Sildenafil has also been reported to attenuate mean pulmonary artery pressure (PAP), pulmonary vascular resistance (PVR), and enhance RV functions by inducing changes in gene expression in heart and lung tissues [55]. Our results confirm these findings; furthermore, as reported in microscopic morphometrical findings, WGLL supplementation demonstrates comparable effects to Sildenafil in alleviating symptoms of pulmonary arterial hypertension.

Pulmonary vascular remodeling is the first and critical component of PAH pathogenesis, with significant involvement of NO/cGMP system. Isoforms of phosphodiesterase enzyme are responsible for hydrolysis of cGMP in pulmonary vasculature [56]. It was previously demonstrated that sildenafil promotes accumulation of cGMP, thus prolonging effects of NO via inhibition of PDE5 enzyme [57]. This study confirms the fact that sildenafil inhibits the elevation of PDE5A expression in right ventricle induced by the disease, affirming previous reports [58]. Upregulation of PDE5 has been reported in conditions like congestive heart failure, pulmonary hypertension and even right ventricular hypertrophy, perhaps as a countering mechanism [59]. Our Western blot findings also verify that in monocrotaline-induced PAH model, expression of PDE5A is highly elevated in the right ventricle. As shown in Figure 3A, WGLL treatment did not affect raised PDE5A levels in the right myocardial tissues, and possibly exerts its protective effects via other signalling pathways. According to our knowledge, this result is unique, since no other research team has studied the impact of *Allium ursinum* extracts on the expression of PDE5 enzyme. Furthermore, levels of PDE5 in lung tissues were elevated after WGLL treatment when compared to the PAH group. Although PDE5A protein levels did not differ between PAH and Control group when measured in lung tissues, this finding does not exclude that the activity of the enzyme may be elevated without a simultaneous rise in its expression. This observation is consistent with previous works, demonstrating that only the phosphorylated form of PDE5 indicates an increase in PDE activity [60,61]. Incidentally, our investigation was limited to the measurement of only the unphosphorylated PDE5 protein levels, therefore, we did not observe any changes in values of Sildenafil-treated group compared to Control group, as reported by others [62]. Although it is well published that elevated levels of PDE5 enzyme are associated with underlying pathological processes of the disease, we observed no significant deterioration in cardiac functions nor histological features of WGLL-treated animals when compared to the PAH group (as seen in Figure 1 and Figure 2 and Table 2), which can be partly explained by the fact that many other signaling pathways are involved in molecular pathogenesis of pulmonary arterial hypertension [63,64,65].

Despite the fact that *Allium sativum* (cultivated garlic) is much more characterized and described in scientific literature, along with previous studies, our recent work suggests that *Allium ursinum* is also a valuable herbal product. Moreover, there are a substantial number of comparative reports in which *Allium ursinum* exerts even more beneficial effects on disease models compared to *Allium sativum* [23,27,66,67]. Last, but not least, Wild garlic is a readily accessible and affordable plant, distributed widely both in Europe and Asia [68].

In summary, Sildenafil exhibited protective efficacy against monocrotalin-induced pulmonary arterial hypertension and vascular remodelling, and beneficial effects exerted by WGLL treatment are well comparable to the standard therapy. Our findings also indicate that it would be feasible to evaluate synergistic actions of Sildenafil and WGLL supplementation in the treatment of pulmonary arterial hypertension.

## 4. Experimental section

### 4.1. Sample Liophyilisation and Mass Spectrometry

Liophylisation of *Allium ursinum* leaves was carried out as previously described by the authors of this present paper [29].

Mass spectrometry (Matrix-assisted laser desorption/ionization–time of flight, MALDI-TOF MS) analyses of the compounds were performed in positive-ion mode using a Bruker Biflex III MALDI-TOF mass spectrometer equipped with delayed-ion extraction (Bruker Daltonics, Bremen, Germany). Desorption/ionization of the sample molecules was effected with a 337-nm nitrogen laser with a pulse width of 3 ns. Spectra from multiple (at least 100) laser shots were summarized using 19 kV accelerating and 20 kV reflectron voltages. External calibration was applied using the [M + Na]^+^ peaks of cyclodextrines DP 6–8 *m*/*z*: 995.31, 1157.36, 1319.41 Da, respectively. The spectrum was obtained from a 2,5-Dihydroxybenzoic acid matrix substance for MALDI-MS, (>99.0% (HPLC), Sigma Matrix) by mixing 10 µL of saturated matrix solution in ethanol:water = 1:1 with 10 µL of sample dissolved in water, then 0.5 µL was applied to the sample target and was allowed to dry at room temperature. The identification of compounds was done on the basis of the mass of [M + H]^+^ or [M + Na]^+^ peaks. The calculations were based on the database of Metabolomics Workbench Data (“The Metabolomics Workbench”, http://www.metabolomicsworkbench.org/).

### 4.2. Study Design

Male rats of the Sprague Dawley strain weighing 200–250 g were purchased from Charles River International Ltd. (Wilmington, MA, USA). Animals were housed under a 12:12 h light-dark cycle in a specific pathogen-free environment and had free access to food and water. Body weights were measured in all animals at the endpoint of the study (8 weeks). PAH was induced by a single subcutaneous injection of monocrotaline (MCT, 60 mg/kg, to the interscapular region), the specific toxic alkaloid of *Crotalaria spectabilis* (Sigma-Aldrich Co., St. Louis, MO, USA) [69], whereas the control group recieved only the vehicle, Dimethyl sulfoxide buffer (DMSO buffer, Control group, *n* = 8). MCT-treated rats were then divided into 3 subgroups as follows: rats that received only MCT injection and developed PAH (PAH group, *n* = 8); rats that received MCT injection and were kept on standard chow enriched with 2% wild garlic leaf liophilizate (WGLL group, *n* = 8); and MCT-injected rats that were treated with 25 mg/kg sildenafil daily via oral (gavage technique) dosing (Sildenafil group, *n* = 8). *Allium ursinum* liophylisate-enriched chow was produced in the laboratory of the Department of Pharmaceutical Technology, University of Debrecen, Hungary. Sildenafil, as well as all other chemicals used in the preparation of buffer solutions for isolated working heart procedure, stains and Western blot technique were obtained from Sigma-Aldrich Co. (Budapest, Hungary). All experimental protocols were approved by the local Ethics Committee of the University of Debrecen (25/2013DEMÁB, 29 January 2014), and the animals received humane care in accordance with the “Principles of Laboratory Animal Care” by EU Directive 2010/63/EU for animal experiments.

### 4.3. Transthoracic Echocardiography (TTE)

**Figure 5:**
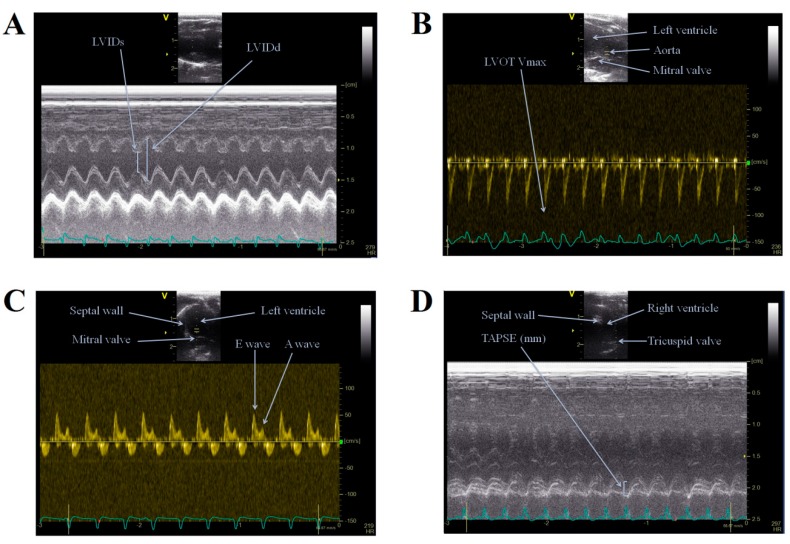
Representative images of echocardiographic examinations of rats in the study. (**A**) M-mode, left ventricle study; (**B**) PW (Doppler) mode, left ventricle outflow tract velocities; (**C**) PW (Doppler) mode, mitral inflow velocities; and (**D**) M-mode, right ventricle study. LVIDs: left ventricular internal diameter in systole; LVIDd: left ventricular internal diameter in diastole; LVOT Vmax: maximal velocity of left ventricle outflow tract; E wave: peak mitral early filling velocity; A wave: peak mitral late (atrial) filling velocity; TAPSE: tricuspid annular plane systolic excursion.

Echocardiographic studies were performed using a Vivid E9 sonograph (GE Healthcare, Little Chalfont, UK) to estimate myocardial functions under light anasthesia with ketamin/xylazine (50/5 mg/kg) intramuscular injection (i.m.) at the start and endpoint of treatments. The thorax was shaved and the animals were positioned in a dorsal decubitus position. Images were obtained using an i13L linear array probe (at 14 MHz) with high temporal and spatial resolution. Complete 2-dimensional, M-mode (at papillary muscle levels), Doppler (PW), and tissue Doppler (TDI) echocardiograms were acquired and digitally stored for further analysis. Doppler imaging at the mitral and aortic valves was obtained from the apical 3- and 4-chamber views. ECG was continously monitored during echocardiographic examinations in all cases. Measurements included interventricular and left ventricular free-wall thickness in diastole and systole (IVSs, IVSd), left ventricular internal diameter at end-diastole (LVIDd) and end-systole (LVIDs), and aortic root diameter (Ao). End-systolic volume (ESV), end-diastolic volume (EDV), stroke volume (SV) and mass of left ventricle (LV mass) were calculated. Fractional shortening was computed by using the equation (LVIDd − LVIDs)/LVIDd × 100%, and the ejection fraction (EF) was calculated by computer using the Teiholz formula. Mitral annular peak systolic excursion (MAPSE) and tricuspidal annular peak systolic excursion (TAPSE) were assessed with M-mode, measuring the distance of mitral or tricuspid annular movement between end-diastole to end-systole. From the mitral inflow velocity image (Doppler), the following measurements were obtained: left ventricular peak E and peak A waves (mitral early and late filling velocities) and the E to A ratio (E/A). Left ventricle outflow tract (LVOT) parameters were also determined: maximal- and mean pressure gradients (LVOT maxPG, LVOT meanPG), as well as maximal- and mean outflow velocities (LVOT Vmax, LVOT Vmean, see Figure 5.). Tissue velocities at the lateral annulus of mitral valve were estimated using spectral tissue Doppler by determining systolic (S’) waves. Visualization of the right ventricle and estimation of systolic function by using TAPSE was accessible in many rats, however, measuring right ventricle diastolic function was unattainable in most cases. All measurements were averaged over three to five consecutive cardiac cycles and were conducted by the same investigator (Bela Juhasz), to avoid interobserver variability. Acquired images were analyzed by using EchoPAC PC softwave (GE Healthcare, Little Chalfont, UK) by two other experienced investigators (Daniel Priksz, Mariann Bombicz). Interobserver variability as well as intraobserver variability was less than 10% in all examinations. The total examination time usually was less than 15 min; thereafter rats were allowed to recover from the procedure [70].

### 4.4. Isolated Heart Parameters

At the endpoint of the study, animals were anesthetized with an intramuscular ketamin/xylazine injection (100/10 mg/kg). A bolus of heparin was administered (1000 U/kg of body weight, intravenously) 15 min before surgery, to avoid thrombosis. Once deep unconsciousness was achieved, hearts were removed by a subxiphoidal incision following thoracotomy, placed in ice-cold buffer, and were quickly cannulated and perfused via the aorta using Langendorff apparatus. The elapsed time between opening the thoracic cavity and onset of perfusion never exceeded 60 s. First, retrograde perfusion was initiated, at a pressure of 100 cm H_2_O for 10 min, which allowed time to wash out the anesthetic. The perfusate was pH 7.4 Krebs-Henseleit bicarbonate buffer, bubbled with Carbogen gas (95% O_2_–5% CO_2_) and containing 118 mM NaCl, 4.7 mM KCl, 1.7 mM CaCl_2_, 25 mM NaHCO_3_, 0.36 mM KH_2_PO_4_, 1.2 mM MgSO_4_, and 10 mM glucose. The left atrium was cannulated during the retrogarde perfusion via the pulmonary vein. A small incision was made at the bifurcation of pulmonary arteries; thus all coronary effluent could be collected by the pulmonary artery. Following Langendorff-perfusion, the system was switched to working mode and hearts were perfused for 15 min. Heart functions, assessed by changes in heart rate and peak systolic pressure development, were monitored throughout the perfusion period using a pressure transducer attached to the aortic outflow line. The following parameters were recorded: aortic pressure (AoP), heart rate (beats/min). Aortic flow (AF, mL/min) and coronary flow (CF, mL/min) were measured by using a flowmeter [71]. All surgeries were performed by the same investigator (Balazs Varga), who was blinded to the treatments, and animals were randomly sacrificed.

### 4.5. Histological Evaluation of Myocardial and Lung Tissue Samples

Following isolated working heart procedure, hearts were dissected transversely at mid-LV level and samples were fixed for 24 h in 4% neutral buffered formalin (pH = 7.4) and further prepared for paraffin sectioning. From the formalin-fixed, paraffin-embedded (FFPE) blocks, 7 μm thick sections were created and stained with hematoxylin-eosin (HE) to visualize tissue architecture [72]. RV hypertrophy (RHV) was assesed on a whole transverse section of the heart and was described as the ratio RV/(LV + interventricular septum (IVS)).

**Figure 6:**
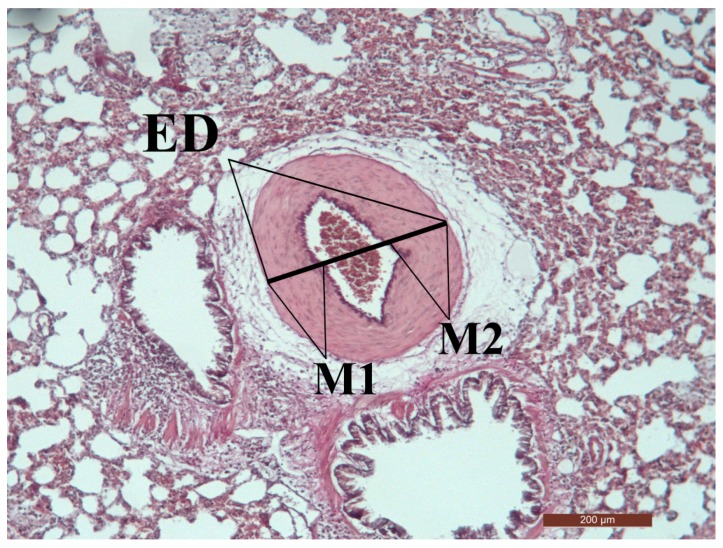
Morphometric analysis of pulmonary arteries in a study rat. The mean external diameter (ED), across the smallest diameter, and the medial wall thickness between the internal (ID) and external elastic lamina of two sides of pulmonary arteries (ED − ID = M1 + M2) were measured. The percentage of medial wall thickness (MWT) was calculated as follows: % MWT = (M1 + M2)/ED × 100 (EVG staining, PAH group, magnification: 100×).

The formalin-fixed, paraffin-embedded left lung sections (7 μm) were stained with HE, and Elastica van Gieson (EVG) for morphometric analysis of vascular dimensions [73]. The external diameters of small pulmonary arteries were measured along the shortest curvature. The relative medial thickness of muscular arteries and lumen-carrying capacity (ranging in size from <50 µm in external diameter) was calculated with the formula ((external diameter − internal diameter)/external diameter × 100) (% MWT = (M1 + M2)/ED × 100) in EVG-stained slides (Figure 6). Fifteen arteriole images per lung section were examined by light microscopy using a Leica DM2500 microscope with DFC 420 camera and Leica Application Suite V3 software (Leica Camera AG, Solms, Germany). The microscope slides were analyzed in a blinded fashion and both the size of the lumen and vessel wall thicknesses were calculated using Scion for Windows Densitometry Image program Version Alpha 4.0.3.2 (Scion Corporation, Frederick, MD, USA).

### 4.6. Western Blot

Deep-frozen rat LV and lung tissue samples were homogenized and then boiled in sample buffer for 10 min. The adjusted final protein concentration was 25 µg mL^−1^. BCA reagent and BSA standard were used for protein quantification assay. The separating gel consisted of 30% glycerol, 6% acrylamide-bis, 0.2 mM Tris, 4  mM Ethylenediaminetetraacetic acid (EDTA) and 0.4% Sodium dodecyl sulfate (SDS) and the polymerization of the gels was induced by ammonium persulfate (10 m/m%) and tetramethylethylenediamine. The upper running buffer was composed of 0.1 mM Tris, 150 mM glycine and 0.1% SDS, and gel was composed of 50 mM Tris, 75 mM glycine and 0.05% SDS. Constant voltage (70 V) was used for separation. A prestained molecular weight standard (ProSieve QuadColor, Lonza; Rockland, MA, USA) was used as loading marker. Separated proteins in the gels were electrophoretically transferred onto nitrocellulose membrane at 380 mA for 45 min. The blotted membrane was blocked with 5% low-fat milk in Tris-buffered saline (TBS) containing 1% Tween 20 (TBS-T buffer). After washing the membrane with TBS-T, primary PDE5A and Glyceraldehyde 3-phosphate dehydrogenase (GAPDH) antibodies (Sigma-Aldrich Co., St. Louis, MO, USA) diluted in TBS-T with 1% low-fat milk were added and incubated overnight at 4 °C. All antibodies were indicated to Western blotting, and were used in dilutions suggested by the manufacturers. The bound antibodies were detected by horseradish peroxidase-conjugated anti-rabbit Ig secondary antibody followed by ECL detection (Western Lightning Plus ECL, PerkinElmer; Waltham, MA, USA) and the signals were recorded by an imaging system (MF-Chemibis 3.2, Central European Biosystems; Budapest, Hungary). Quantitative analysis of scanned blots was carried out using the Scion for Windows Densitometry Image program Version Alpha 4.0.3.2 (Scion Corporation). Signal intensity for bands corresponding to each protein of interest was estimated and reported in arbitrary units ± SEM [74].

### 4.7. Data Analysis and Statistical Procedures

All data are presented as the average magnitudes of each outcome in a group ± standard error of the mean (SEM). Statistical analysis was performed using one-way analysis of variance (ANOVA) with Bonferroni post-testing (when normality test was passed) or by Kruskal–Wallis test with Dunn’s post-testing (if the normality test was not passed). Statistical analyses were conducted using GraphPad Prism software for Windows, version 5.01 (GraphPad Software Inc., La Jolla, CA, USA). Probability values (*p*) less than 0.05 were considered statistically significant.

## 5. Conclusions

Our recent paper highlights the possible protective effects of *Allium ursinum* lyophilisate supplementation in pulmonary arterial hypertension investigated on a rat model of the disease. According to our knowledge, this is the first study to evaluate possible benefits of *Allium ursinum* in a model of PAH, and to directly compare it with a well-known PDE inhibitor, sildenafil. Our findings demonstrate that WGLL supplementation exerts protection against MCT-induced pulmonary arterial hypertension in a rat model, shown by significant changes in either echocardiographic and isolated heart functions, or histological analyses. Right-heart systolic functions assessed by echocardiography showed improvements in tricuspid annular plane systolic excursions, while isolated heart experiments revealed an increase in left ventricle aortic flow and cardiac output. These findings were further supported by Western blot analyses, where WGLL supplementation did not show any changes in PDE5A expression levels when compared to values measured in samples harvested from PAH animals. In contrast, histology revealed that increase in medial wall thickness caused by monocrotaline-treatment was counteracted by WGLL treatment. Our hypothesis is partly confirmed experimentally, as supplementation with WGLL, a low-cost and reasonable natural product, suppresses the symptoms of MCT-induced pulmonary hypertension in a rat model, but its protective effects on cardiovascular system seem to be independent from the PDE5 pathway; thus underlying mechanisms need further investigations.

## Figures and Tables

**Table 1:** Mass spectrometric analysis of the Wild garlic (*Allium ursinum*) samples. Table 1 shows measured and exact *m*/*z* values of the main compounds present in *A. ursinum* leaf liophylisate.

Structure	PubChem CID	Common Name	Input *m*/*z*	Exact *m*/*z*	Δ	Formula	Ion
74640	24211973	Kaempferol-3-*O*-rutinoside	617.4	617.1472	0.2528	C_27_H_30_O_15_Na	[M + Na]^+^
74640	24211973	Kaempferol-3-*O*-rutinoside	633.3	633.1212	0.1788	C_27_H_30_O_15_K	[M + K]^+^
46189	5280459	Quercitrin	471.2	471.0898	0.1102	C_21_H_20_O_11_Na	[M + Na]^+^
46189	5280459	Quercitrin	487.2	487.0637	0.1363	C_21_H_20_O_11_K	[M + K]^+^
47726	5748554	Juglanin	441.4	441.0792	0.3208	C_20_H_18_O_10_Na	[M + Na]^+^
47726	5748554	Juglanin	457.3	457.0531	0.2469	C_20_H_18_O_10_K	[M + K]^+^
68247	160270	Dracorubin	527.2	527.1255	0.0745	C_32_H_24_O_5_K	[M + K]^+^
68247	160270	Dracorubin	511.2	511.1516	0.0484	C_32_H_24_O_5_Na	[M + Na]^+^
48987	11289628	Blumeatin	341.2	341.0422	0.1578	C_16_H_14_O_6_K	[M + K]^+^
48987	11289628	Blumeatin	325.2	325.0683	0.1317	C_16_H_14_O_6_Na	[M + Na]^+^

**Table 2:** Echocardiographic data of the four rat test groups in the study. Right ventricle function was estimated using tricuspid annular plane systolic excursion (TAPSE). Significant decreases were found in TAPSE values of PAH (pulmonary arterial hypertension) group animals compared to the Controls. Values for animals receiving sildenafil injection and Wild garlic (*Allium ursinum*) liophylisate-enriched chow (WGLL) were elevated in comparison to the PAH group.

Parameter	Control	PAH	Sildenafil	WGLL
LV Ejection Fraction (%)	73.39 ± 3.638	82.61 ± 2.911	76.93 ± 2.294	76.13 ± 2.327
LV Fractional Shortening (%)	38.64 ± 3.268	47.75 ± 3.432	40.84 ± 2.177	40.49 ± 2.059
LV mass (g)	1.388 ± 0.085	1.343 ± 0.037	1.487 ± 0.036	1.469 ± 0.057
Stroke volume (mL)	0.459 ± 0.070	0.456 ± 0.056	0.445 ± 0.081	0.452 ± 0.032
HR (bpm)	269.5 ± 16.21	274.2 ± 9.37	264.3 ± 10.39	243.1 ± 9.76
LVOT maxPG (mmHg)	2.225 ± 0.247	2.415 ± 0.260	2.401 ± 0.381	2.128 ± 0.179
LVOT meanPG (mmHg)	1.032 ± 0.112	1.067 ± 0.096	1.169 ± 0.185	0.876 ± 0.074
LVOT Vmax (m/s)	0.753 ± 0.041	0.746 ± 0.445	0.748 ± 0.082	0.720 ± 0.032
LVOT Vmean (m/s)	0.432 ± 0.025	0.451 ± 0.018	0.463 ± 0.045	0.398 ± 0.017
Lat S’ (cm/s)	32.29 ± 1.782	40.51 ± 2.710	50.39 ± 2.278 *	39.36 ± 3.565
MV E vel (cm/s)	65.81 ± 3.860	64.71 ± 3.046	58.09 ± 2.906	63.56 ± 2.484
MV A vel (cm/s)	40.15 ± 4.253	37.54 ± 5.484	30.82 ± 1.054	39.19 ± 2.385
MV E/A ratio	1.841 ± 0.167	1.856 ± 0.165	1.903 ± 0.052	1.745 ± 0.129
MAPSE (mm)	2.085 ± 0.089	1.961 ± 0.098	1.905 ± 0.057	1.944 ± 0.150
TAPSE (mm)	2.308 ± 0.074	1.697 ± 0.098 *	2.390 ± 0.069 ^#^	2.021 ± 0.071 ^#^

* *p* < 0.05 compared to Control; # *p* < 0.05 compared to PAH.
